# Autophagy and Skin Diseases

**DOI:** 10.3389/fphar.2022.844756

**Published:** 2022-02-18

**Authors:** Kim Klapan, Dagmar Simon, Alexander Karaulov, Marina Gomzikova, Albert Rizvanov, Shida Yousefi, Hans-Uwe Simon

**Affiliations:** ^1^ Institute of Pharmacology, University of Bern, Bern, Switzerland; ^2^ Department of Dermatology, Inselspital, Bern University Hospital, University of Bern, Bern, Switzerland; ^3^ Department of Clinical Immunology and Allergology, Sechenov University, Moscow, Russia; ^4^ Laboratory of Molecular Immunology, Institute of Fundamental Medicine and Biology, Kazan Federal University, Kazan, Russia; ^5^ Institute of Biochemistry, Brandenburg Medical School, Neuruppin, Germany

**Keywords:** atopic dermatitis, autophagy, inflammation, psoriasis, skin cancer

## Abstract

Autophagy is a highly conserved lysosomal degradation system that involves the creation of autophagosomes, which eventually fuse with lysosomes and breakdown misfolded proteins and damaged organelles with their enzymes. Autophagy is widely known for its function in cellular homeostasis under physiological and pathological settings. Defects in autophagy have been implicated in the pathophysiology of a variety of human diseases. The new line of evidence suggests that autophagy is inextricably linked to skin disorders. This review summarizes the principles behind autophagy and highlights current findings of autophagy’s role in skin disorders and strategies for therapeutic modulation.

## Introduction

**FIGURE 1:**
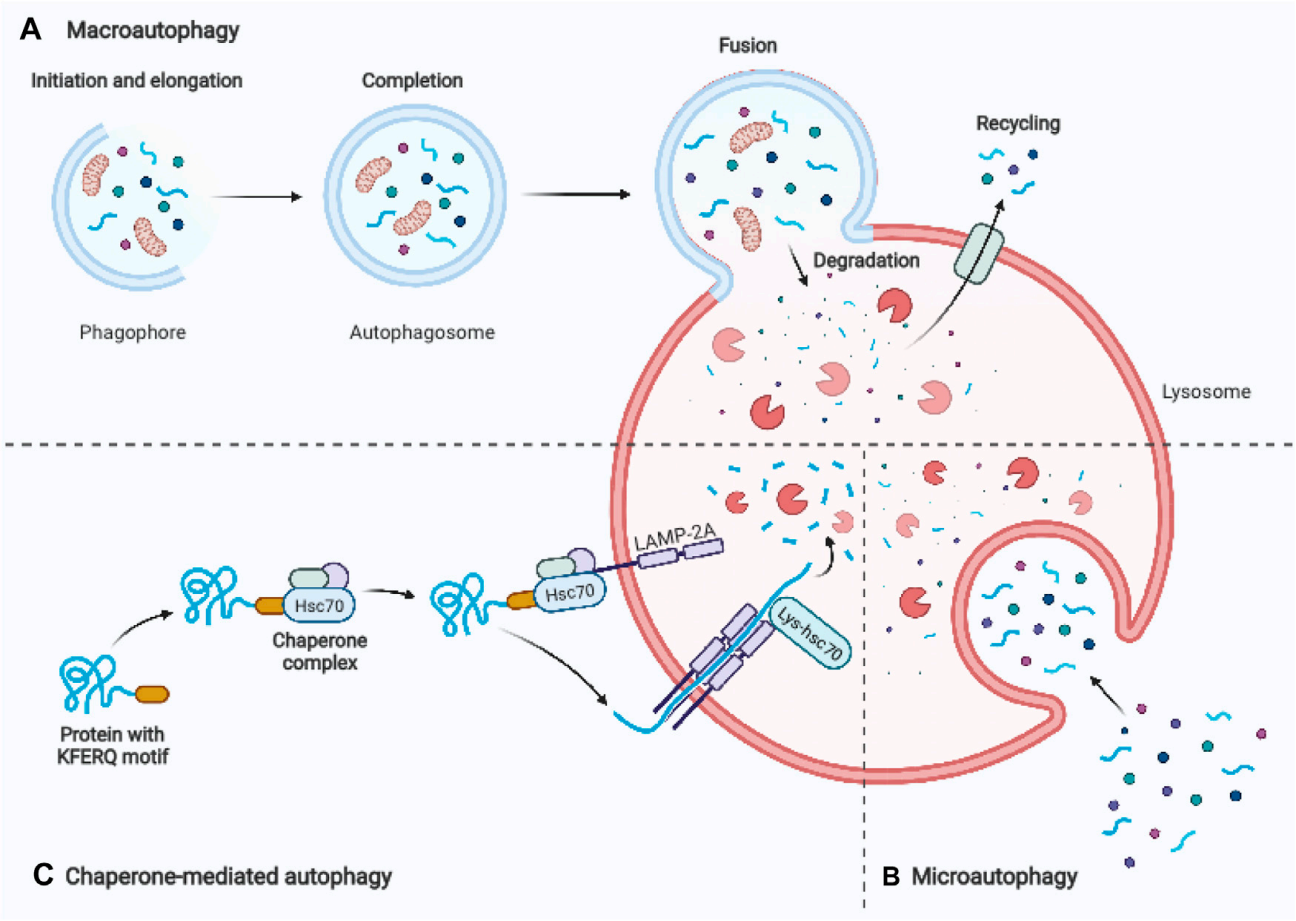
Schematic representation of three main autophagy pathways. **(A)** Macroautophagy: An autophagosome is formed by enclosing a section of cytoplasm, including organelles, in an isolation membrane known as phagophore. The autophagosome’s outer membrane merges the lysosome, and the autolysosome degrades the inside material. **(B)** Microautophagy: Inward invagination of the lysosomal or late endosomal membrane directly engulfs small fragments of cytoplasm. **(C)** Chaperone-mediated autophagy: Hsc70 and cochaperones in the cytosol identify substrate proteins with a KFERQ-like pentapeptide sequence. After binding to lysosomal LAMP-2A, they are transferred into the lysosomal lumen. Adapted from biorender.com.

Autophagy is a conserved lysosomal degradation pathway that eukaryotic cells utilize to regulate their homeostasis (Simon et al., 2017). In 1963, Christian de Duve, the discoverer of lysosomes, was first to coin the name “autophagy.” The term derives from ancient Greek meaning “self-eating” (Klionsky, 2008). One significant advance in understanding the molecular mechanism of autophagy was the identification of autophagy-related genes in yeast. Yoshinori Ohsumi’s laboratory recently identified important autophagy-related proteins (ATGs) that are required for cargo transport to the vacuole in yeast, for which he was awarded the Nobel Prize in Physiology or Medicine in 2016 (Dikic and Elazar, 2018). Today, we distinguish macroautophagy, microautophagy, and chaperon-mediated autophagy based on the distinct mechanisms of cargo delivery to lysosomes. Macroautophagy is the most common type of autophagy, which involves the formation of an isolation membrane, dubbed a phagophore, that sequesters a small section of the cytoplasm, including organelles, to create a double membrane vesicle called an autophagosome (Figure 1A). The autophagosome merges with the lysosome to form an autolysosome, where lysosomal hydrolases degrade the infused cargo (Mizushima and Komatsu, 2011). Microautophagy is a process that utilizes the invagination of the lysosomal membrane to sequester cytoplasm (Schuck, 2020) (Figure 1B). Unlike the other two processes, chaperone-mediated autophagy does not involve membrane reorganization, namely, it includes particular chaperone complexes translocating the targeted proteins across the lysosomal membrane. Unfolded proteins are carried into the lysosomal lumen by a multimeric translocation complex by the transmembrane protein LAMP-2A, which is an isoform of LAMP-2 (Cuervo and Wong, 2014) (Figure 1C). Macroautophagy is thought to be the major type of autophagy, therefore in this review, we will simply refer to it as “autophagy.”

Autophagy’s primary functions are to maintain the normal turnover of intracellular proteins and organelles, to produce amino acids in times of nutrient scarcity, and to regress retired tissues (Levine and Yuan, 2005). Therefore, autophagy is a mechanism that maintains the homeostasis of individual cells. Additionally, studies over the last two decades have established that autophagy is required for a variety of immune system functions, including pathogen clearance (Wild et al., 2011), antigen presentation (Lee et al., 2010; Li et al., 2011; Mintern et al., 2015), immune cell development (O’Sullivan et al., 2016), and modulation of inflammatory responses (Dikic and Elazar, 2018). Any disruption of the autophagic process is likely to cause, or at the very least contribute to the pathophysiology of a disease, for example, in neurodegenerative diseases and in cancer, where it is dangerously exposed to misuse by contributing to tumor progression and therapy resistance (Mizushima and Levine, 2020). In this review, we will summarize the process of autophagy and provide an overview of the involvement of autophagy in major skin diseases.

## Molecular Machinery of Autophagy

**FIGURE 2:**
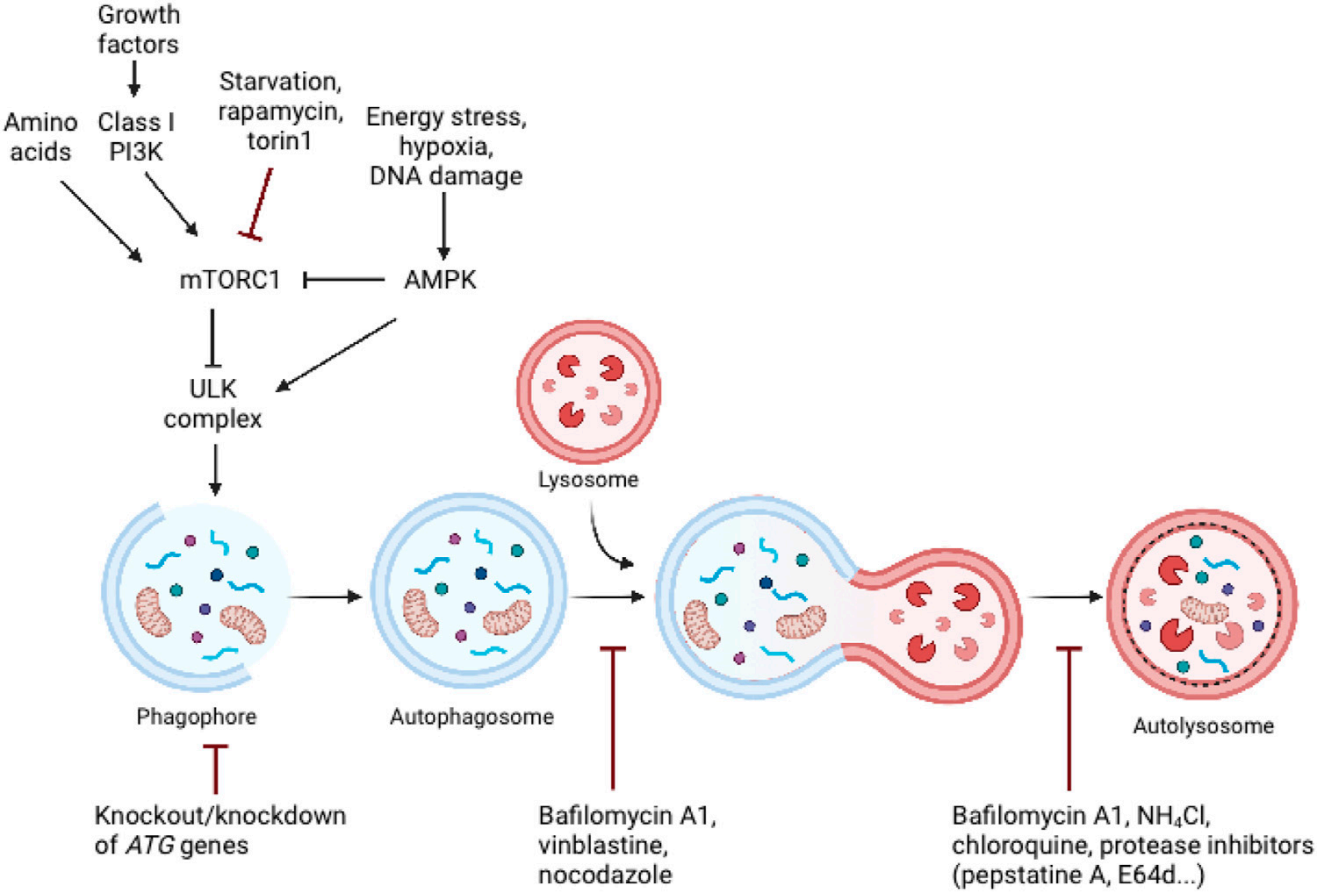
Schematic representation of major signalling pathways that regulate autophagy and the inhibition of autophagy by genetic and pharmacological approaches. Autophagy is controlled by a variety of growth and nutrition signaling pathways. mTORC1 activity, which reflects cellular nutritional status, is a major regulator of autophagy. Since the ULK complex is inactivated by mTORC1 activity, sufficient amounts of amino acids and growth hormones prevent autophagy. Cellular stress, such as energy deprivation, DNA damage, and hypoxia inhibit mTORC1 activity, resulting in the release and activation of the ULK complex, which triggers autophagosome formation. Adapted from biorender.com.

The autophagic process is tightly regulated by the ATG protein family. Autophagy’s central machinery is composed of multiple steps, starting with the induction of the autophagic process, followed by vesicle nucleation and expansion, and concluding with the formation of the autolysosome where delivered cargo is degarded (Chen and Klionsky, 2011). Mammalian target of rapamycin (mTOR) is the catalytic subunit of two distinct complexes, called mTOR Complex 1 (mTORC1) and 2 (mTORC2). mTORC1 has been shown to be a signaling hub that regulates cell growth and nutritional status, as well as promotes the synthesis of proteins, ribosome biogenesis, nutrient transport, and lipid synthesis (Benjamin et al., 2011). Additionally, it plays a role in regulating the autophagic process’s catabolic activity (Benjamin et al., 2011) (Figure 2). A significant inducer of autophagy is a deficiency of amino acids, which results in the suppression of mTORC1 activity and activation of the inhibited ULK complex (Dikic and Elazar, 2018). mTORC1 entails mTOR, the complex’s catalytic kinase component, and subunits raptor, PRAS40, mLST8, and Deptor that control its activity and substrate availability (Zoncu et al., 2011). Investigations into the mechanism of action of rapamycin, a macrolide inhibitor of mTOR generated by bacteria with a wide range of clinical applications such as an antifungal, immunosuppressant, and anti-cancer medication, have yielded several insights into mTOR signaling (Sehgal, 2003). Rapamycin is frequently employed in research investigations to determine the involvement of mTORC1 in a particular process because to its presumed potency and selectivity. Rapamycin engages with the immunophilin FKBP12 in mammalian cells, and the resulting FKBP12–rapamycin complex then binds to the mTOR FRB domain. By an as-yet-unidentified mechanism, the FKBP12–rapamycin complex reduces mTORC1 kinase activity allosterically (Benjamin et al., 2011; Thoreen et al., 2012). On the other hand, the novel mTOR inhibitors are ATP analogues that compete with ATP for binding to the kinase domain in mTOR, inhibiting mTOR kinase activity. ATP-competitive mTOR inhibitors, such as Torin1, significantly degrade protein synthesis and proliferation compared to rapamycin, owing to their suppression of rapamycin-resistant mTORC1 activities (Thoreen et al., 2012). Rapamycin is unable to completely inhibit the function of mTORC1 and has a negligible effect on the mTORC2 in the majority of cell types (Guertin and Sabatini, 2007), whereas Torin1 inhibits mTORC1 and mTORC2 (Benjamin et al., 2011; Thoreen et al., 2012).

Furthermore, autophagy can also be initiated in response to diminishing cellular energy levels, such as those associated with glucose deprivation, as detected by cell homeostasis regulatory kinases 5′ AMP-activated protein kinase (AMPK) (Liu and Sabatini, 2020). Additionally, stressful conditions such as hypoxia or DNA damage induce AMPK activation, which promotes autophagy via mTORC1 inhibition or direct phosphorylation of the ULK complex (Dikic and Elazar, 2018). Namely, low cellular energy (high AMP/ATP ratio) triggers the activation of AMPK. Activated AMPK suppresses energy-intensive processes, such as protein synthesis, and promotes ATP-generating processes, such as fatty acid oxidation (Wullschleger et al., 2006). Activated AMPK inhibits mTORC1 in two ways: indirectly through activation of the upstream mTORC1 negative regulator TSC2, or directly through phosphorylation and inactivation of raptor (Shaw et al., 2004; Gwinn et al., 2008).

Inhibition of mTOR has been shown to have a wide range of inhibitory effects on immunological effector cells. Rapamycin suppresses dendritic cell (DC) development and function (Hackstein et al., 2003), as well as T-cell proliferation (Mondino and Mueller, 2007), which is the mechanism through which it exerts its immunosuppressive effect. Cancer is caused by defects in growth control, therefore it's unsurprising that mTOR also plays a role in its pathogenesis. Despite the potential benefits of mTOR inhibition as an antitumor therapy, as single-agent therapy rapalogues have failed to deliver robust, broad anticancer effect in clinical trials (Benjamin et al., 2011). There could be various explanations for rapalogues’ poor performance. The repressed negative feedback loop induced by mTORC1 inhibition stimulates PI3K–AKT signaling and may potentially increase cancer cell survival, as AKT activates an anti-apoptotic response (O’Reilly et al., 2006). Rapamycin is mainly inefficient at suppressing mTORC2 activity; this activity is a component of the PI3K–mTORC2–AKT signaling axis, which is hyperactive in a variety of tumors (Benjamin et al., 2011).

The ULK complex, which is composed of the protein kinase ULK1/2, ATG13, ATG101, and FIP200 is formed during autophagy induction, originating from locations where ATG9 vesicles align with the endoplasmic reticulum (ER) (Feng and Klionsky, 2017). Once ULK complex is activated, it stimulates the recruitment and activation of the class III phosphatidylinositol-3-kinase (PI3K) complex that is comprised of Beclin-1, ATG14, VPS15, and the lipid kinase VPS34 (Proikas-Cezanne et al., 2015). Two ubiquitin-like conjugation mechanisms are required for effective phagophore membrane elongation and closure. Conjugation of the main autophagy proteins ATG12, ATG5, and ATG16L leads to the formation of the complex ATG12-ATG5-ATG16L. On the expanding phagophore membrane, the ATG12-ATG5-ATG16L1 complex together with ATG7 and ATG3 stimulates the conjugation of microtubule-associated protein light chain 3 (LC3) with phosphatidylethanolamine (PE) forming lipidated LC3-II (Mizushima and Levine, 2020). LC3-II has been considered a marker of autophagy due to its presence on the autophagosomal membrane and the variations in its cellular level during the autophagic process (Tanida et al., 2005; Tanida et al., 2008). The newly formed autophagosomes fuse with lysosomes to form autolysosomes, where lysosomal hydrolytic enzymes degrade both the internalized cargo and the autophagosomal membrane. Amino acids and other degraded components are excreted into the cytosol and reused (Mizushima and Komatsu, 2011). Protein p62 is one of the most well-characterized selective autophagy substrates. p62 is a cellular protein that is widely expressed in animals but not in plants or fungi (Johansen and Lamark, 2011). Through its association with LC3, p62 binds to ubiquitin and transports protein aggregates to autophagosomes. Since p62 is constantly degraded by autophagy, autophagy dysfunction is accompanied by p62 accumulation (Komatsu and Ichimura, 2010; Liu et al., 2016).

## Autophagy in Infectious Skin Diseases

**FIGURE 3:**
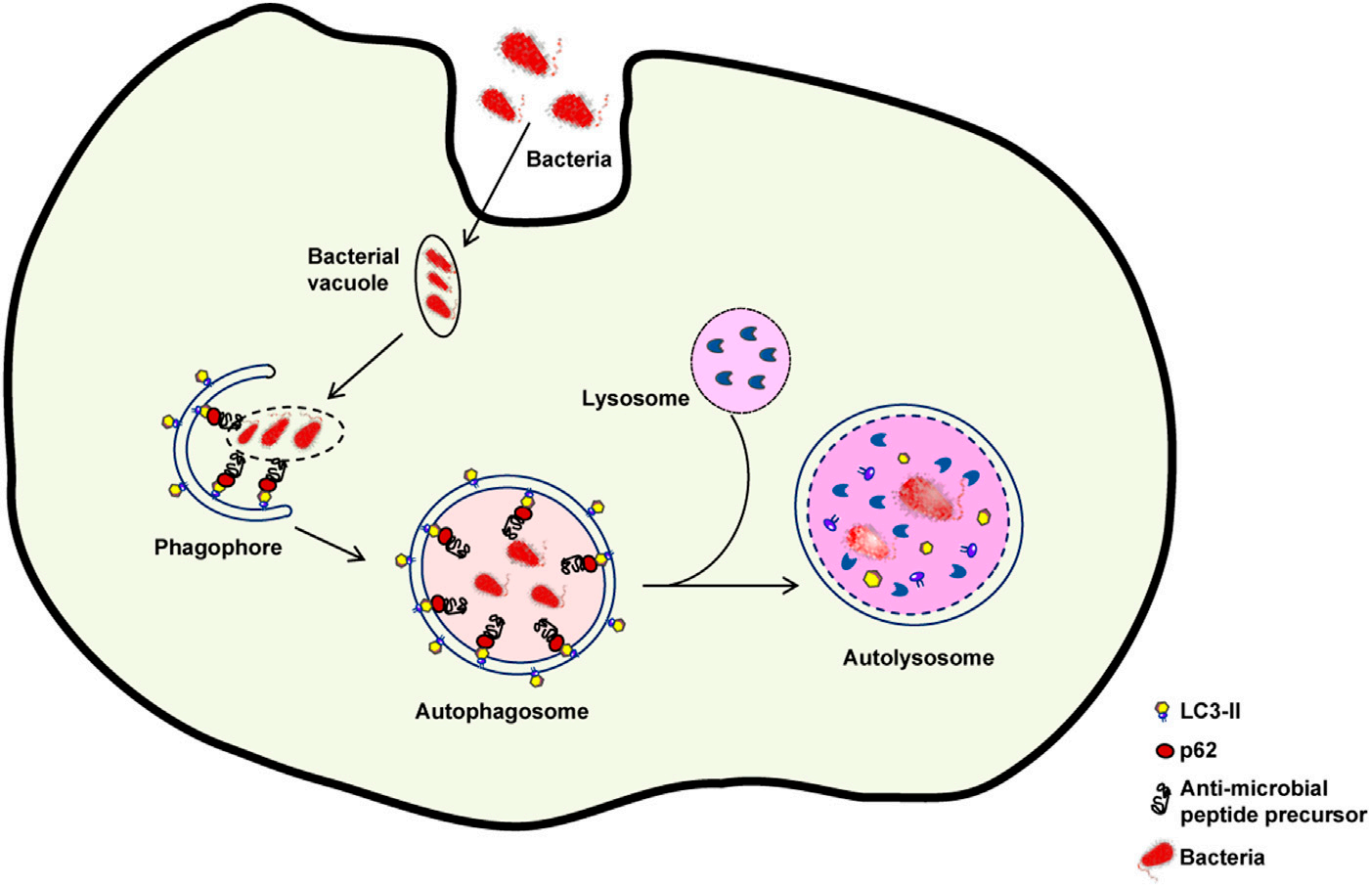
Xenophagy: An intracellular defence mechanism against bacteria. Following host cell invasion, vacuoles harboring intracellular bacteria merge with autophagosomes and engage the autophagy machinery to kill the pathogenes in autolysosomes. However, several bacteria have developed mechanisms to escape xenophagy. For example, some bacteria are able to decrease the fusion of bacteria-containing vacuoles with autophagosomes or of autophagosomes with lysosomes. If the autophagic process is inhibited, bacterial reproduction can occur.

The skin plays a key function in defending the organism against foreign pathogens through a variety of processes. One of such processes, dubbed xenophagy, is devoted to the eradication of invading microbes (Sharma et al., 2018). Autophagy sequesters intracellular pathogens such as *Salmonella*, *Listeria*, *Mycobacteria*, *Legionella*, *Shigella*, and group A *Streptococcus* via xenophagy (Gutierrez et al., 2004; Nakagawa et al., 2004; Amer and Swanson, 2005; Casanova, 2017) (Figure 3). Thus, xenophagy is critical to the innate immune response because it not only eliminates invasive microorganisms but also stimulates the production of host defense peptides and initiates the adaptive immune response by presenting antigens (Gutierrez et al., 2004; Deretic et al., 2013).

***Staphylococcus aureus*** is a major cause of skin infection and is frequently isolated from the skin of patients with atopic dermatitis during flares (Geoghegan et al., 2018). Schnaith et al. (2007) found the autophagy induction is required for *Staphylococcus aureus* replication, cytoplasmic escape, and host cell death. Several investigations have established that *Staphylococcus aureus* is capable of parasitizing both professional and non-professional phagocytes by modulating the host autophagy pathway in order to establish an intracellular survival niche (Vozza et al., 2021). *Staphylococcus aureus* is rapidly ubiquitinated intracellularly and then recognized by autophagy receptors in non-professional phagocytes. These receptors associate ubiquitin with microorganisms via the LC3, trapping bacteria in the autophagosome (Neumann et al., 2016). The beneficial impact of autophagy is further demonstrated by the autophagy protein ATG16L that mediates a unique mode of protection against *Staphylococcus aureus* infection. Keller et al. (2020) demonstrated that ATG16L1 and other ATG proteins protect against alpha-toxin, a factor released by *Staphylococcus aureus*, by releasing ADAM10 from exosomes-extracellular vesicles derived from endosomes. ATG16L1 deficiency exacerbates the mortality caused by *Staphylococcus aureus* in mice. Furthermore, by maintaining tolerance for the alpha-toxin, autophagy may protect host cells against *Staphylococcus aureus* infection. Inhibition of autophagy resulted in an increase in cell death triggered by the alpha-toxin in mouse endothelial cells, indicating that autophagy serves as a barrier for cells to maintain membrane homeostasis under stressful circumstances (Maurer et al., 2015). *Staphylococcus aureus*, on the other hand, has evolved ways to circumvent the autophagy pathway. It has been demonstrated that the alpha-toxin inhibits the fusion of autophagosomes and lysosomes, hence preventing *Staphylococcus aureus* breakdown before it reaches the cytoplasm in an ovary hamster cells (Mestre et al., 2010). Moreover, The autophagosomes containing *Staphylococcus aureus* lacked both acidification and acquisition of lysosome-associated membrane protein-2 (LAMP-2), a hallmark for late endosomes and lysosomes. This abnormal autophagic response was also found in bovine mammary epithelial cells treated with *Staphylococcus aureus* (Wang et al., 2019). *Staphylococcus aureus* utilizes metabolic activation of autophagy to ensure its intracellular survival. To be specific, Bravo-Santano et al. found that *Staphylococcus aureus* severely reduced glucose and amino acid pools in order to elicit a starvation response, which results in highly active glutamine in host cells to meet their own metabolic requirements. These modifications induce autophagy via the AMP-activated protein kinase (AMPK) and extracellular signal-regulated kinase (ERK) signaling pathways (Bravo-Santano et al., 2018).

**Group A Streptococci** (GAS), a Gram-positive bacteria that frequently colonize the skin’s surface, can cause a variety of ailments, including skin infections, such as erysipelas, impetigo, cellulitis, scarlet fever, necrotizing fascitis, and streptococcal toxic shock syndrome (Cohen-Poradosu and Kasper, 2007; Henningham et al., 2012). The autophagic process functions as a defensive mechanism against intracellular pathogens, demonstrating that autophagy is a critical innate immunity mechanism. For example, Nakagawa et al. have reported that within HeLa cells, autophagic machinery might effectively remove pathogenic GAS (Nakagawa et al., 2004). GAS became engulfed by autophagosome-like compartments of HeLa after egressing from endosomes into the cytoplasm and were destroyed following fusion of these compartments with lysosomes (Nakagawa et al., 2004). On the other hand, GAS survived, proliferated, and were released from autophagy-deficient Atg5^−/−^ cells (Nakagawa et al., 2004). Furthermore, it was demonstrated that GAS serotype M1T1 can avoid autophagy and reproduce rapidly in the cytoplasm of human HEp-2 epithelial cells (Barnett et al., 2013). SpeB, a streptococcal cysteine protease, is required for this process, as SpeB destroys ubiquitylation components such as p62, NDP52, and NBR1 both *in vitro* and in the host cell cytoplasm (Barnett et al., 2013). Most recently it has been demonstrated that FBXO2, a glycoprotein-specific substrate receptor in the SKP1/CUL1/F-box protein ubiquitin ligase complex, recognizes the GlcNAc side chains on the GAS surface carbohydrate structure and stimulates ubiquitin-mediated xenophagy against GAS (Yamada et al., 2021).

**Herpes simplex viruses** 1 and 2 (HSV-1 and HSV-2), as well as varicella zoster virus, are classified as human-herpesviruses. HSV-1 infection results in corneal keratitis and cold sores in the orolabial region, whereas HSV-2 infection primarily results in genital lesions (Shukla and Spear, 2001). HSV likes to inhibit the cell’s self-destruction processes—whether autophagy in the lysosome or apoptosis in the mitochondria, in order to continue parasitizing the cell for survival (Banerjee et al., 2020). It has been reported that HSV-1 replication is reduced in chemically mutagenized mouse L fibroblasts with elevated baseline autophagy levels (Le Sage and Banfield, 2012). Adult brains are protected from viral encephalitis by autophagy activation, however this protection is age-dependent, since it appears to be harmful and promote apoptosis in newborn mouse brains (Wilcox et al., 2015). Moreover, HSV-1 evolved ways to disrupt the regulation of autophagy. Each herpesvirus genome has been characterized as being capable of encoding multiple anti-autophagic proteins that act at various phases of autophagy. For instance, the HSV-1 neurovirulence protein ICP34.5 interacts with Beclin-1 to limit autophagy by impairing PKR and eIF2 phosphorylation in mice neurons, all of which are essential for autophagy induction (Orvedahl et al., 2007). Therefore, inhibition of autophagy is a unique biochemical mechanism by which viruses circumvent innate immunity and induce disease (Orvedahl et al., 2007). Along with ICP34.5, the Us11 protein, which is produced later in HSV-1 infection than ICP34.5, has been shown to block autophagy by direct interaction with PKR (Lussignol et al., 2013).

Furthermore, Lee et al. (2010) have discovered that silencing the *ATG5* gene also reduces HSV-2 antigen processing and presentation on MHC class II molecules, hence increasing susceptibility to HSV-2 infection *in vivo*. According to the newest study, the conserved autophagy receptor optineurin (OPTN) is required for neuronal survival during possibly deadly CNS HSV infections (Ames et al., 2021). When confronted with HSV-1, Optn-deficient mice exhibit severe cognitive impairment and an increased vulnerability to fatal CNS infection (Ames et al., 2021). Multiple studies reveal several techniques adopted by Herpesviruses to evade the degradative process, but it remains unknown whether this cellular mechanism plays a significant role in viral infection resistance.

**Human papillomaviruses** (HPVs) are non-enveloped, double-stranded DNA viruses that have a strong preference for epithelial cells (Mattoscio et al., 2018). HPVs are connected with a wide variety of conditions, ranging from benign verrucae vulgares and condylomata acuminata to cervix, vulva, anus, and penis cancers (Mattoscio et al., 2018). As is the case with many other viruses, HPV manipulates the autophagic process to increase its replication within host-infected cells. There are several studies demonstrating that E5, E6, and E7 oncoproteins acquired distinct methods to impact the host autophagic pathway in order to cause cellular transformation emphasizes the critical role of autophagy throughout the viral-mediated cancer process. For instance, HPV16 E5 ectopic expression in an HPV-negative keratinocytic cell line decreased levels of LC3-II, inhibited degradation of p62, and decreased the quantity of autophagosomes in keratinocyte growth factor and serum-starved stimulated cells, indicating autophagosome formation failure (Belleudi et al., 2015). It was demonstrated that HPV16 E5 affects the transcriptional activation of the autophagic machinery by decreasing the mRNA levels of important autophagic genes such as *Beclin 1*, *ATG5*, *LC3*, *ULK1*, *ULK2*, *ATG4a*, and *ATG7*, implying an impairment of phagophore formation (Belleudi et al., 2015). Furthermore, it has been reported that activation of Akt and mTOR occurs several minutes after human keratinocytes are exposed to HPV type 16. Activation of the PI3K/Akt/mTOR signaling pathway inhibited autophagy during the initial stages of virus-host cell interaction, thereby promoting virus infection (Surviladze et al., 2013). In line with that, impairing autophagy with the early autophagy inhibitor 3-methyladenine (3-MA) or by genetically silencing crucial autophagic genes in primary human keratinocytes significantly enhances HPV16 infection in human keratinocytes, highlighting the critical role of host autophagy in regulating the early steps of the HPV lifecycle (Griffin et al., 2013). Recent research has shown that HPV-positive oropharyngeal squamous cell carcinoma cells exhibit decreased autophagic activity, which is mediated by the ability of HPV-E7 to engage with AMBRA1, compete for its binding to Beclin-1, and activate its calpain-dependent degradation (Antonioli et al., 2021).

***Candida albicans*** is a dimorphic commensal fungus that colonizes healthy human skin, mucosa, and the reproductive system. However, it can also be an opportunistic fungal pathogen, resulting in clinical presentations such as disseminated candidiasis and chronic mucocutaneous candidiasis (Kashem and Kaplan, 2016). Autophagy has been found to be critical in regulating the spread of fungal infection and disease susceptibility (Kanayama and Shinohara, 2016). In an *in vivo* murine *Candida albicans* infection model, autophagy proteins have been demonstrated to have a protective function in host defense (Nicola et al., 2012; Kanayama et al., 2015), however some publications have also suggested that autophagy proteins are not required for human host protection in *Candida albicans* infection (Rosentul et al., 2014; Smeekens et al., 2014). It was reported that knocking down *ATG5* in J774.16 murine macrophages reduced LC3 recruitment to *Candida albicans*-containing phagosomes, and mice with ATG5 deficiency in myeloid cells, such as macrophages, DCs, and neutrophils, were more susceptible to *Candida albicans* (Nicola et al., 2012). Another study validates the role of autophagy in *Candida albicans* host defense in mice and gives more insight on the mechanism by which macrophages play a role in this process (Kanayama et al., 2015). When Kanayama et al. employed *Candida albicans* to infect ATG7 deficient mice in myeloid cells, they found that these mice had a lower survival rate and a higher fungal load in the spleen and kidney than wild type mice (Kanayama et al., 2015). Furthermore, active autophagy enabled vaginal epithelial cells to resist the damage produced by *Candida albicans* infection, whereas cells with impaired autophagy succumbed to the infection (Shroff and Reddy, 2018). This indicates that autophagy is crucial for the survival of human vaginal epithelial cells during *Candida albicans* infection (Shroff and Reddy, 2018). Although numerous studies have been conducted on the role of autophagy in *Candida albicans* infection, additional research is necessary to understand the effect of autophagy on host immunity against *Candida* in humans.

## Autophagy in Inflammatory and Autoimmune Skin Diseases

Inadequate pathogen and dead cell clearance eventually lead to tissue inflammation. Autophagy as a non-inflammatory mechanism eliminates pathogens and dead cells. As a result, autophagy abnormalities can contribute to inflammation as well as potentially initiate or worsen the autoimmune disease (Yang et al., 2015). The earliest evidence that autophagy may have a role in inflammation regulation came from a genome-wide association studies (GWAS) on patients with Crohn’s disease, a gastrointestinal inflammatory condition, in which single nucleotide polymorphism (SNP) in the autophagy-related gene *ATG16L1* were found to have a substantial correlation with disease susceptibility (Hampe et al., 2007). There has been an increase in the study into autophagy and autoimmune skin disorders since the discovery of the association between autophagy and autoimmune diseases.

**FIGURE 4:**
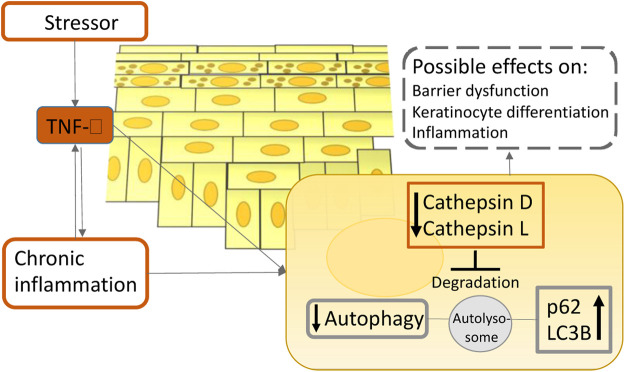
A simplified scheme illustrates the role of TNF-α on autophagy in atopic dermatitis and psoriasis pathogenesis. Upon inflammation in the epidermis, TNF-α reduces the enzymatic activity of lysosomal cathepsins, leading to lysosomal dysfunction which ultimately results in autophagy suppression. This process could contribute to the chronicity of cutaneous inflammatory disorders.

**Psoriasis** is a chronic inflammatory autoimmune disease charcterized by sharply delineated erythematous patches with silvery scales (Boehncke and Schon, 2015). A cytokine-led intricate inflammatory cascade leads to infiltration of immune cells into the lesional skin, excessive keratinocyte proliferation, and increased expression of endothelial adhesion molecules and angiogenic mediators (Nestle et al., 2009). Until recently very little was known about the role of autophagy in psoriasis. One of the earliest studies of autophagy in psoriasis revealed that a polymorphism in the autophagy gene *ATG16L1* can be used to predict the likelihood of acquiring psoriasis (Douroudis et al., 2012). Overly stimulated immune system and keratinocyte hyperproliferation are one of the main characteristics of psoriasis (Armstrong and Read, 2020). In one of the early publications on the function of autophagy in psoriasis, it was discovered that autophagy abnormalities cause inflammatory cytokine production and cell proliferation in keratinocytes (Lee et al., 2011), highlighting an important role of autophagy in psoriasis pathogenesis. Lee’s group has revealed evidence that TLR2/6 or TLR4 stimulation triggers the autophagy pathway and increases p62 expression in primary human keratinocytes. Furthermore, inhibiting autophagy enhances inflammatory cytokine production and p62 expression in primary human keratinocytes. Moreover, suppressing *hp62* via RNA interference significantly decreases NF-κB activation, inflammatory cytokine production, cathelicidin expression, and keratinocyte proliferation. This study shed new light on the roles of autophagy and p62 in cutaneous inflammation management (Lee et al., 2011). Given that p62 participates in a variety of different biological processes, including ubiquitination (Sanchez-Martin and Komatsu, 2018), these results should not be taken at face value. Additionally, overexpression of p62 does not suggest autophagy induction alone; it imply autophagy inhibition as well, which should be verified by concurrent upregulation of LC3-II (Komatsu and Ichimura, 2010). On the other hand, according to newer data, it is becoming more evident that autophagy is impaired in psoriasis, implying its role in the pathogenesis of the disease. Namely, Wang et al. have discovered that inactivating the mitogen-activated protein kinase (MAPK) family decreases keratinocyte autophagy, which is positively linked with psoriatic severity in patients and mice models (Wang et al., 2020). Additionally, reducing autophagic flux alleviated psoriasiform inflammation. Furthermore, they have found that an autophagy-dependent unconventional secretory pathway (autosecretion) involving ATG5 and golgi reassembly stacking protein 2 (GORASP2) increases psoriasiform keratinocyte inflammation (Wang et al., 2020). Moreover, our group has recently demonstrated the evidence that the long exposure of pro-inflammatory cytokine TNF-α on primary epidermal keratinocytes decreases the levels of major cathepsins in lysosomes, which consequently impairs autophagy. This finding was also validated in psoriasis patients’ specimens (Klapan et al., 2021) (Figure 4). In agreement with our report, several other reports suggest that psoriasis-associated cytokines such as TNF-α and IL-17A impair autophagy in primary keratinocytes (Varshney and Saini, 2018; Tang et al., 2021). In support of the idea that the autophagic process is inhibited in psoriasis, evidence-based therapies for psoriasis such as retinoids, vitamin D analogs, and UVB phototherapy, were shown to induce autophagy (Hoyer-Hansen et al., 2005; Rajawat et al., 2010; Yang et al., 2012; Qiang et al., 2013). Thus far, research suggests that autophagy dysfunction contributes to the pathophysiology of psoriasis. Therefore, pharmaceuticals approved for use in other illnesses that induce autophagy could be useful as psoriasis therapies.

**Systemic lupus erythematosus (SLE)** is a chronic autoimmune illness with a wide range of severity and course. It is characterized by a proclivity for flare-ups (Maidhof and Hilas, 2012). The interaction of genes and environmental factors results in a variety of immunologic changes, culminating in permanent immune responses to autologous nucleic acids. Autoantibodies and immune complex depositions induce tissue damage in the kidneys, lungs, heart, muscles, vessels, central nervous system, skin, and joints, resulting in considerable morbidity and mortality (Fanouriakis et al., 2021). Over 100 loci related to SLE susceptibility have been identified through GWAS. *ATG5*, *CDKN1B*, *DRAM1*, *CLEC16A*, and *ATG16L2* are five autophagy-related genes that were reported to be associated with SLE risk (Molineros et al., 2017). These findings provided compelling evidence that autophagy plays a significant role in the genetic etiology of SLE (Molineros et al., 2017). Moreover, it was reported that the ATG5 rs573775 allele appears to have an effect on SLE susceptibility, cytokine production, and disease characteristics, depending on other factors such as functional IL-10 genotype (Lopez et al., 2013; Kamel et al., 2020). Furthermore, numerous studies have established a relationship between SNP in the *ATG5* gene and the Prdm1–ATG5 intergenic region with the development of SLE and, in a European population, rheumatoid arthritis (International Consortium for Systemic Lupus Erythematosus et al., 2008; Gateva et al., 2009; Raychaudhuri et al., 2009). Moreover, ATG genes such as *mTOR*, *Beclin-1*, *LC3*, and *p62* were discovered to be expressed differently by lupus peripheral blood mononuclear cells (Wu et al., 2017). Autophagy-mediated B cell regulation can directly alter the pathophysiology of SLE because B cells are a crucial actor in SLE, functioning on both antibody-dependent and independent processes. Interestingly, autophagy has been found to be enhanced in lupus B and T cells (Clarke et al., 2015; Wu and Adamopoulos, 2017; Jang et al., 2021). It has been reported that blocking macrophage autophagy alleviates activated lymphocyte-derived DNA-induced lupus in mice, perhaps through inhibiting the generation of proinflammatory cytokines such as IL-6 and TNF-α (Li et al., 2014). Today, autophagy has been identified as a therapeutic target in SLE treatment. Increasing clinical outcomes attested an autophagy inducer rapamycin enables the alleviation of disease, organs protection, and the extension of life span (Fernandez et al., 2006; Lai et al., 2013; Oaks et al., 2016; Eriksson et al., 2019). Several lupus medications have the potential to function as autophagy regulators, but their effects on the autophagy process appear counterintuitive. Rapamycin can be used to decrease hyperactive mTORC1 signaling, which is a characteristic of both T and B cells in autoimmunity. In a mouse model of lupus, rapamycin alleviated nephritis and increased IL-2 production (Warner et al., 1994). Lai et al. (2018) reported on a 12-month treatment with rapamycin of 29 patients with active SLE who were resistant to or intolerant to conventional medicines. The data demonstrate that a progressive improvement in disease activity is associated with a correction of pro-inflammatory T-cell lineage specification in patients with active SLE who received rapamycin for 12 months. Additionally, several other medications have an influence on autophagy. The autophagy suppressor hydroxychloroquine (HCQ) is one of the most often used medications for SLE (Dorner, 2010). In individuals with SLE, HCQ is used alone or in combination with steroids and immunosuppressive medicines to prolong patients’ lives by lowering lupus flares and organ damage accumulation (Nirk et al., 2020). Case studies have demonstrated that HCQ medication increases long-term survival in people with SLE, whereas those who are not treated with HCQ have an increased risk of severe SLE exacerbations (James et al., 2007; Willis et al., 2012). Therefore, it appears that both autophagy inducers and inhibitors have a postive impact on SLE treatment. This could be owing to the fact that diseases progress at various rates or because autophagy modulators exhibit distinct effects on different cell types.

**Vitiligo** is a pigmentary condition of the skin and mucous membranes that is characterized by patchy loss of skin pigmentation caused by melanocyte loss. Vitiligo is a multifactorial disorder with several etiological explanations, including autoimmune, oxidative stress, and hereditary (Bergqvist and Ezzedine, 2020). According to the newest research, induction of autophagy protects against intrinsic metabolic stress and seeks to counteract degenerative processes in normal-appearing vitiligo skin, where melanocytes and fibroblasts are already susceptible to premature senescence (Bastonini et al., 2021). Furthermore, cohort research conducted in Korea discovered a connection between non-segmental vitiligo and gene polymorphisms associated with UV resistance (Jeong et al., 2010). By controlling melanosome degradation in keratinocytes, autophagy plays a critical role in skin color determination, contributing to ethnic variability in skin color. Melanin levels were considerably lowered by autophagy activators and elevated by autophagy inhibitors in human specimens, as well as *in vitro* human skin substitutes (Murase et al., 2013). Singh et al. (2021) discovered that TNF-α exposure increased ATG12 and Beclin-1 mRNA levels after 12 h, and subsequently induced apoptosis in human melanocytes after 48 h. Their findings reveal a functional relationship between autophagy and the demise of melanocytes. Moreover, IL-17 promotes autophagic cell death by inducing a cellular stress microenvironment in melanocytes. The autophagy-mediated apoptotic pathway is enhanced by IL-17, which contributes to vitiligo pathogenesis (Zhou et al., 2018). Recent evidence suggests that ATG7-dependent autophagy is required for melanocyte redox homeostasis and biological processes such as melanogenesis, proliferation, apoptosis, and senescence, particularly under conditions of oxidative stress (Qiao et al., 2020).

**Atopic dermatitis** is a prevalent chronic inflammatory skin disorder affecting primarily children in developed countries. It is defined by a compromised epidermal barrier and an overactive immune system (Simon et al., 2019). Diverse epidermal changes have been observed in both lesional and non-lesional skin of patients with AD, including increased permeability and transepidermal water loss, an imbalance in protease and protease inhibitor expression, increased pH, decreased levels of tight junction proteins, and increased susceptibility to infection (Bieber, 2008). Autophagy regulates host defensive systems against invading pathogens like *Staphylococcus aureus*, which has a role in the etiology of atopic dermatitis (Simon et al., 2019). Additionally, the autophagic process is involved in the regulation of inflammation and keratinocyte differentiation (Akinduro et al., 2016; Qian et al., 2017). Furthermore, our group has showed that patients suffering from atopic dermatitis have notably decreased levels of major lysosomal cathepsins when compared to healthy controls, indicating the importance of autophagy in its pathogenesis (Klapan et al., 2021) (Figure 4). Thus, pharmacological modification of autophagic activity may be a therapy option for atopic dermatitis. Namely, Kwon et al. have published that a new prospective treatment for atopic dermatitis, a moisturizer containing pentasodium tetracarboxymethyl palmitoyl dipeptide-12 (PTPD-12), a known autophagy stimulator, has been demonstrated to considerably enhance skin barrier function and pruritus in patients with atopic dermatitis (Kwon et al., 2019). Therefore, future studies should focus on modulating autophagy activity in atopic dermatitis patients as a new potential therapeutic approach.

## Autophagy in Skin Cancer

Autophagy in cancer cells has an ambiguous meaning since it can operate as a tumor- suppressor during the early stages of carcinogenesis by digesting potentially hazardous substances or damaged organelles, thus preventing the spread of damage, including DNA changes. However, autophagy is a tumor-promoting mechanism in advanced phases of tumor formation due to its ability to maintain tumor viability in stressful microenvironments (Amaravadi et al., 2019; Chavez-Dominguez et al., 2020). Within this setting, it is becoming increasingly obvious that modulating autophagy may improve therapy outcomes in advanced cancer patients (Levy et al., 2017).

**Skin squamous cell carcinoma (SCC)** is a frequent type of skin cancer that originates in the thin, flat squamous cells that comprise the skin’s outer layer (Burton et al., 2016). Although a variety of variables can contribute to an increased risk of SCC, cumulative sun exposure, particularly during infancy and adolescence, is critical to its development and the incidence of this type of skin cancer is anticipated to continue to rise until 2040 (Gurney and Newlands, 2014; Combalia and Carrera, 2020). The significance of autophagy and the processes behind it in SCC still remain unknown. SCC has been reported to have a high level of autophagy, with increased autophagy activity being related to tumor aggressiveness (Yu et al., 2015). Furthermore, Sivridis et al. (2011) have established a link between autophagy activity and the aggressiveness of SCC as measured by tumor thickness and proliferation. Namely, autophagic activity in SCC could be potentially used to predict tumor aggressiveness when expressed as high LC3A/“stone-like structures” ratios. An emerging line of evidence is indicating that inhibiting autophagy may be a promising method for increasing tumor cells’ susceptibility to anticancer treatment. Thus, chloroquine’s (CQ) suppression of autophagy may be a possible SCC treatment (Verschooten et al., 2012; Ou et al., 2019). Moreover, inhibition of autophagy by 3-MA in SCC improved the effect of 5-fluorouracil-induced chemotherapeutic sensitivity, suggesting that, indeed, modifying autophagy may be a beneficial therapeutic strategy (Zhang et al., 2015). In line with this, in methotrexate (MTX)-treated human SCC, the tumor suppressor WW domain-containing oxidoreductase (WWOX) inhibits autophagy in order to induce apoptosis. Thus, by inhibiting autophagy, WWOX confers susceptibility on SCC cells to MTX-induced apoptosis, while failure to increase WWOX expression confers chemotherapeutic drug resistance. As a result, increasing WWOX levels in cancer cells may be a potential method of suppressing autophagy (Tsai et al., 2013). Additionally, the most recent finding has demonstrated that inhibiting autophagy via silencing human histocompatibility leukocyte antigen complex P5 (HCP5) decreased the enhancer of zeste homolog 2 (EZ2H) expression and inhibited the STAT3/VEGFR2 pathway via competitive binding to miR-138-5p, which resulted in apoptosis in SCC cells (Zou et al., 2021).

**FIGURE 5:**
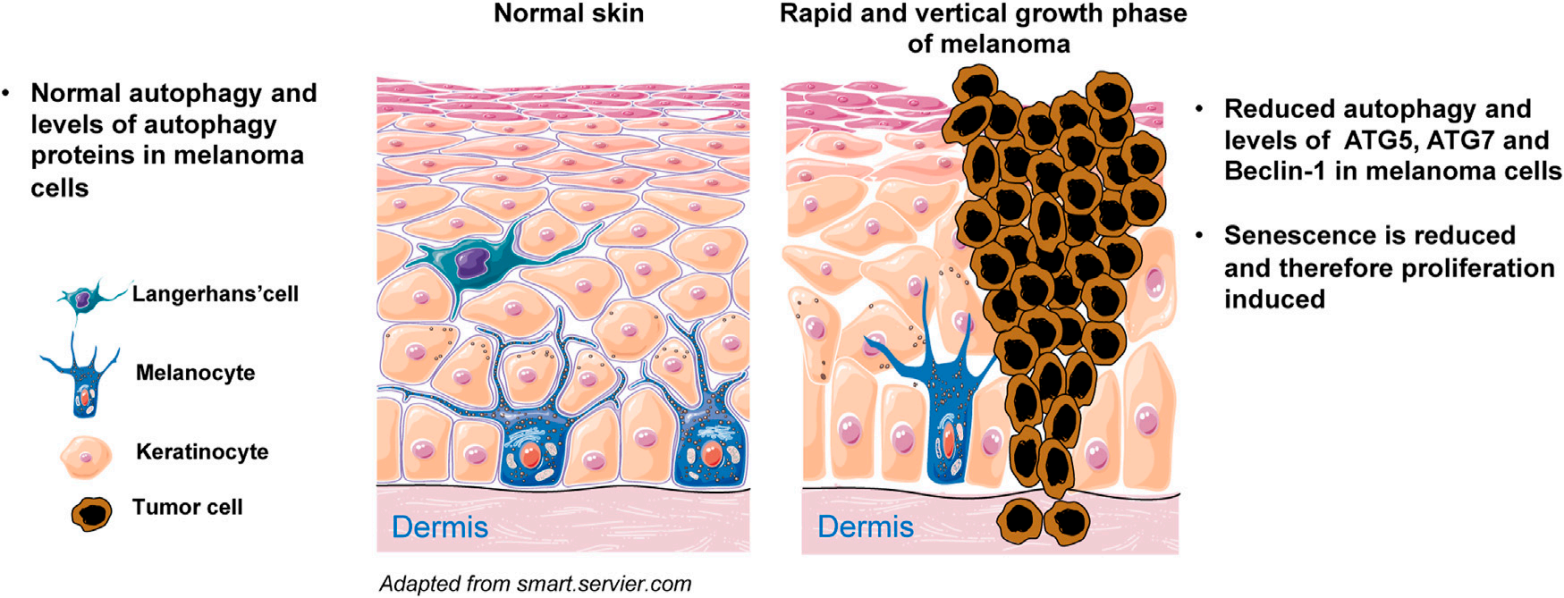
Simplified scheme of melanoma initiation. Melanocytes are normally regulated by epidermal keratinocytes and produce physiological levels of ATGs and autophagy. Melanocytes can proliferate abnormally in response to oncogenic mutations and cluster along the basement membrane of the epidermis. Cells experience limited proliferation at this stage and eventually enter senescence. Senescence does not occur when autophagy is diminished, for example owing to reduced expression of ATG5, ATG7 and/or Beclin-1. Under such conditions, melanocytes rapidly proliferate and the melanoma demonstrates vertical growth into both epidermis and dermis.

**Melanoma** is a malignancy of melanocytes and is the most aggressive form of skin cancer. Melanoma formation necessitates a complex interplay of external and endogenous factors, such as sun exposure and distinct genetic alterations (Schadendorf et al., 2015). Autophagy appears to have a complicated and dynamic role that is highly dependent on the disease’s progression stage, metabolic demand, intrinsic, and extrinsic factors. It has been found that the transformation of melanocytes to malignant melanoma cells is accompanied by changes in autophagy activity (Levine and Kroemer, 2008; Rosenfeldt and Ryan, 2011). Autophagy’s role in skin cancer is debatable: there has been evidence of both tumor-suppressive and tumor-promoting properties, mostly depending on tumor stage. Various autophagy-related genes and proteins have been identified to be altered during melanomagenesis, suggesting that they could be used as a predictor of patients’ survival, invasiveness, and/or treatment sensitivity in individuals with melanoma (Lazova et al., 2012; Liu et al., 2013; Tang et al., 2016; Frangez et al., 2021). Studies examining the changes in the autophagic state of cells during melanomagenesis discovered that BRAF mutations do not initiate the vast majority of melanomas but rather represent a progression stage of cancer (Dong et al., 2003). Autophagy’s tumor-suppressive activity appears to be inhibited during the early stages of tumor growth. For example, several studies have demonstrated that autophagy markers such as LC3 and Beclin-1 levels were changed in melanocytic neoplasms, resulting in severely impaired autophagy during the early phases of melanocyte malignant transformation (Miracco et al., 2010; Hara and Nakamura, 2012; Maes et al., 2014). Our group has published a study demonstrating that during the early phases of malignant transformation, autophagy is diminished in comparison to normal melanocytes, which is associated with a decrease in ATG5 expression (Liu et al., 2013). Additionally, decreased ATG5 levels in primary melanomas compared to benign nevi were significantly linked with poor progression-free survival in a group of patients with early stage cutaneous melanoma (Liu et al., 2013) (Figure 5). Furthermore, it has been reported that mice heterozygous for the Beclin-1null allele, as well as mice with constitutional ATG5 or ATG7 impairment, all of which are critical mediators of autophagy, develop spontaneous tumors (Mathew et al., 2009; Takamura et al., 2011). Other data, on the other hand, indicate that the autophagic process is increased in already established melanoma, allowing cancer cells to persist despite high metabolic demands and a harsh microenvironment. For instance, Hara and Nakamura (2012) reported that autophagy activity is elevated in metastatic melanoma, as evidenced by Beclin-1 overexpression. Reduced p62 expression is indicative of active autophagy, as seen in advanced stage melanomas, where autophagy is frequently stimulated to improve tumor survival (Klionsky et al., 2016). Furthermore, the presence of an autophagic phenotype in all studied melanoma specimens, as determined by LC3 levels, implies that a relationship between autophagy and cancer invasion is crucial in the evolution of malignant melanoma. The high level of autophagy expression shows that autophagy is critical for melanoma cell survival and that invasive and metastatic cells may be sensitive to anti-autophagic therapy (Lazova et al., 2010). Moreover, Beclin-1 and LC3 have been found to be overexpressed in advanced or metastatic melanoma as compared to early primary lesions, and have also been linked to cell proliferation markers like Ki67 expression (Miracco et al., 2010; Hara and Nakamura, 2012; Lazova et al., 2012; Maes et al., 2014). Interestingly, under stressful conditions, melanoma cells have been demonstrated to increase autophagy activity as a protective strategy; Marino et al. (2012) discovered that melanoma cells cultivated in acidic environments can survive by increasing autophagy activity. Autophagy inhibition with *ATG5* silencing lowered melanoma cell survival in acidic environments. Thus, modulating autophagy activity may be beneficial in the treatment of melanoma patients. Indeed, it has been shown that inhibiting autophagy in combination with an mTOR or BRAF inhibitor exhibits strong anticancer efficacy (Ma et al., 2014; Rangwala et al., 2014). Recently, phase I/II clinical trials with HCQ-mediated autophagy suppression have been conducted in a variety of malignancies, including melanoma, and myeloma (Vogl et al., 2014). On the other hand, a population-based cohort study of patients with connective tissue diseases was carried out to assess the effect of HCQ and CQ on the risk of acquiring cancer. In this study, long-term exposure to HCQ/CQ did not reduce the risk of cancer. However, HCQ/CQ may reduce the risk of metastatic cancer and mortality. Therefore, using HCQ or CQ in the treatment of malignancies may have a beneficial effect for patients (Fardet et al., 2017). Furthermore, the mTORC1 inhibitor temsirolimus triggered pro-survival autophagy, and the combination of temsirolimus with CQ treatment considerably enhanced therapeutic response (Xie et al., 2013). Therefore, inhibiting autophagy is thought to be an effective technique for sensitizing malignant cells to current melanoma therapy. On the other hand, autophagy activation may be considered a therapeutic technique when autophagic cell death induction is required as a secondary mode of cell death in apoptotic melanoma cells.

## Conclusion

A growing body of evidence indicates that autophagy plays a significant role in the pathogenesis of a variety of skin disorders. The autophagic machinery may serve as an innate defense mechanism against pathogen invasion, such as *Staphylococcus aureus* and GAS. Autophagy appears to be dysfunctional in autoimmune and inflammatory skin diseases, suggesting that including autophagy modulators (Table 1) into existing therapy may be of high value, for example activating autophagy with autophagy inducers may aid in the treatment of psoriasis and atopic dermatitis. Based on current evidence, the autophagic process plays a dual role in skin cancer, as autophagy deficiency promotes tumor progression, while functional autophagy permits cancer cells to survive under conditions of stress, possibly contributing to treatment resistance. Additional research, including investigations of human cells and tissues, is necessary to identify pharmacological targets for modulating the autophagic pathway as a preventive or therapeutic intervention for skin diseases.

**TABLE 1:** Overview of autophagy modulators in skin diseases.

Disease	Autophagy inducer/inhibitor	Experimental system	Outcome	References
Atopic dermatitis	Pentasodium tetracarboxymethyl palmitoyl dipeptide-12	Patients with mild-to-moderate atopic dermatitis	SCORAD score and skin hydration improvement	Kwon et al. (2019)
Psoriasis	HCQ	Monocyte-derived Langerhans cells and monocyte-derived dendritic cells	Induction of the Th17 cytokines IL-23 and IL-6 production and release	Said et al. (2014)
SLE	Rapamycin	Lupus mice	Prolongation of the survival of mice	Ramos-Barron et al. (2007)
	Rapamycin	Patients with active SLE	Correction of pro-inflammatory T cell lineage	Lai et al. (2018)
	HCQ	Patients with stable SLE	Reduced clinical flare-up, reduction of anti-DNA autoantibodies and normalization of the complement activity	Canadian Hydroxychloroquine Study Group (1991), Monzavi et al. (2018), Wakiya et al. (2020)
SCC	Saracatinib	Head and neck squamous cell carcinoma (HNSCC) cell line	Supression of HNSCC growth and cell cycle progression	Ammer et al. (2009)
	Cetuximab	Advanced HNSCC patients	Statistically significant improvement in patients’ survival	Burtness et al. (2005), Vermorken et al. (2008)
Melanoma	HCQ	Patients with either advanced solid tumors or advanced melanoma	High rate of disease stabilization in patients with advanced cancer	Rangwala et al. (2014)
	Rapamycin			
*Staphylococcus aureus* infection	Selenium	Mouse macrophages	Alleviation of the blockade of autophagic flow, reduction of the transcription of MAPK and NF-κB signalling pathways, and inhibition of the proliferation of *S. aureus*	Zang et al. (2020)
